# Simultaneous path weak-measurements in neutron interferometry

**DOI:** 10.1038/s41598-024-76167-6

**Published:** 2024-10-29

**Authors:** Armin Danner, Ismaele V. Masiello, Andreas Dvorak, Wenzel Kersten, Hartmut Lemmel, Richard Wagner, Yuji Hasegawa

**Affiliations:** 1 Atominstitut, TU Wien, Stadionallee 2, 1020 Vienna, Austria; 2 Institut Laue-Langevin, 71 avenue des Martyrs, 38000 Grenoble, France; 3 Laboratoire Léon Brillouin, UMR12 CEA-CNRS, Université Paris-Saclay, CEA Saclay, 91191 Gif sur Yvette, France; 4 Department of Applied Physics, Hokkaido University, Kita-ku, Sapporo 060-8628 Japan

**Keywords:** Matter waves and particle beams, Quantum mechanics, Qubits, Single photons and quantum effects

## Abstract

The statistical properties of the detection events constituting the interference fringes at the output of an interferometer are well-known. Nevertheless, there is still no unified view of what is happening to a quantum system inside an interferometer. Strong measurements of path operators destroy the interference effect. In weak measurements, an observable is weakly coupled to a pointer system and the resulting weak values quantify the observable by minimally disturbing the system. Previous which-way experiments with weak measurements could extract either the real or imaginary part of a single weak value with each ensemble. Here, we present the simultaneous full complex quantification of two path weak values with a single ensemble in a Mach–Zehnder neutron interferometer. Magnetic fields, oscillating with different frequencies, change the energy state in each interferometer path. The time-dependent phase between the energy states distinctly marks each path. The resulting beating intensity modulation at the interferometer output gives both path weak values. For the present experiment, the weak values’ absolute value and phase directly describe the observed amplitude and phase of the intensity modulation.

## Introduction

The double-slit experiment and interferometry as a whole are corner-stones of quantum physics. Any coherent system, e.g., light, electrons or the neutrons regarded in this letter, may exhibit interference fringes at the detector behind an interferometer. This is accurately described as a wave phenomenon^1^. While the first interference experiments with light^2^ and electrons^3^ worked with high-flux sources, the same effects also emerge with single photons^4^, electrons^5^, and neutrons^6^. The latter self-interference defies any classical analysis.

Notwithstanding the accurate description through delocalised waves^7^, one may attempt to find out which path the system went. One may, e.g., block a particular path of the interferometer—only to see the interference fringes disappear completely. Such a strong measurement with a beam blocker destroys the investigated interference phenomenon. The behaviour of the interference fringes was investigated with deterministic and probabilistic absorbers for neutrons^8^. This characterised the coherence of the quantum state dependent on the kind of absorption and different interaction strengths.

A tool to quantify the system inside an interferometer while preserving interference are weak measurements^9^ where the interaction between the quantum system to be measured and the measurement instrument is so weak that the back-action of the measurement is practically negligible. Weak measurements are defined for a pre- and post-selection procedure. A specific state is prepared (pre-selected) and another state is accepted (post-selected) towards the detector. A degree of freedom (DOF) of the system is investigated. A pointer system, i.e., auxiliary DOF, is manipulated through a weak interaction implemented midway between pre- and post-selection. Through entanglement between the two DOF, the interaction marks the investigated DOF. Comparing the detected intensities with and without applying the weak interaction quantifies the investigated DOF through weak values. Usually, the real and imaginary parts of a weak value are measured separately. In the interpretation of their physical significance, this separation is maintained: the real part gives the best estimate of an observable^10,11^ and the imaginary part is a measure of the intrinsic measurement disturbance on the system^11,12^.

A realisation of a weak measurement with photons^13^ was implemented early after the theoretical introduction. Among the wide spectrum of applications, weak measurements were utilised, e.g., for signal amplification^14,15^ and proposals for quantum smoothing^16,17^. With neutrons, the path DOF was used as a pointer system to retrieve spin weak values^18^ and, vice versa, the spin degree of freedom to retrieve path weak values^19,20^. A photonic proposal^21^ suggested multiple simultaneous path markings through the energy DOF via mirrors with different vibration frequencies. The proposal was realised with photons^22^ and in an adopted version with neutrons^23^. The experimental intensity at the output of the interferometer was time-resolved and exhibited oscillations in time with the applied frequencies. A path marking through the energy DOF as a pointer was demonstrated. However, weak values were not retrieved. In photonic setups, successive measurements of weak values were reported^24–27^. For atoms, the weak measurements of the polarisation and spin were reported^28,29^.

In this work, we want to find out which path the neutrons went in a two-path neutron interferometer^30–33^. Therefore, the path DOF is investigated with the goal of retrieving both path weak values simultaneously. Each previously determined real and imaginary part of a path weak value was extracted separately with a different ensemble. In the case of weak measurements, it was regularly stressed that the weakness of the interaction minimises the disturbance on the system^34,35^. Thus, each weak value describes the intermediate quantum system between pre- and postselection as best as possible. But it is not clear if multiple weak values extracted with different ensembles are valid for each ensemble.

In the present experiment, we exploit the minimal disturbance of the measurement to accomplish a simultaneous weak measurement of two path observables. The energy DOF, i.e., the system of different energy levels, is used as pointer system for the path DOF. In each path, the energy is manipulated differently to trace each path individually with a characteristic time-dependent phase. Detecting the resulting time-dependent intensity makes both path weak values accessible simultaneously. The validity of the retrieved weak values for the whole ensemble is hence assured. The measurements are carried out in parallel in the interferometer in contrast to the reported successive measurements.

## Results

### Theory

The pre-selected (initial) state ${|{\textrm{i}}\rangle }$ of a neutron in the interferometer is written as

1 $$\begin{aligned} {|{\textrm{i}}\rangle }=\frac{1}{\sqrt{2}}\big ({|{\textrm{I}}\rangle }+\textrm{e}^{-\textrm{i}\chi }{|{\textrm{II}}\rangle }\big ){|{\uparrow _z}\rangle }, \end{aligned}$$

where $\chi$ is the relative phase between paths I and II. The spin state is written with up and down arrows with the index of the basis used (*x*, *y*, *z*). The post-selected (final) state ${|{\textrm{f}}\rangle }$ is written as

2 $$\begin{aligned} {|{\textrm{f}}\rangle }=\frac{1}{\sqrt{2}}\big ({|{\textrm{I}}\rangle }+{|{\textrm{II}}\rangle }\big ){|{\uparrow _x}\rangle }. \end{aligned}$$

The path weak values^9^${\langle {{\hat{\Pi }}_j}\rangle }_\textrm{w}={\langle {\textrm{f}|{\hat{\Pi }}_j|\textrm{i}}\rangle }/{\langle {\textrm{f}|\textrm{i}}\rangle }$ of the path operators $\hat{\Pi }_j={|{j}\rangle }{\langle {j}|}$, $j\in \{\textrm{I}, \textrm{II}\}$, are calculated as

3 $$\begin{aligned} {\langle {{\hat{\Pi }}_\textrm{I}}\rangle }_\textrm{w}=\frac{1}{1+\textrm{e}^{-\textrm{i}\chi }},\ \ {\langle {{\hat{\Pi }}_\textrm{II}}\rangle }_\textrm{w}=\frac{1}{1+\textrm{e}^{\textrm{i}\chi }}. \end{aligned}$$

Note that these weak values are the same as for the initial and final states ${|{\mathrm {i'}}\rangle }=\big ({|{\textrm{I}}\rangle }+\textrm{exp}(-\textrm{i} \chi ){|{\textrm{II}}\rangle }\big )/\sqrt{2}$ and ${|{\mathrm {f'}}\rangle }= \big ({|{\textrm{I}}\rangle }+{|{\textrm{II}}\rangle }\big )/\sqrt{2}$, without the spin component, since ${|{\textrm{i}}\rangle }$ and ${|{\textrm{f}}\rangle }$ are spin-path separable. Thus, the results are valid for the weak-value extraction of both sets of initial and final states. In the polar representation

4 $$\begin{aligned} {\langle {{\hat{\Pi }}_j}\rangle }_\textrm{w}=A_j \textrm{e}^{\textrm{i}\varphi _j}, \end{aligned}$$

the absolute value $A_j$ and the phase $\varphi _j$ are calculated as

5 $$\begin{aligned} A_j=\frac{1}{2\cos (\chi /2)},\ \ \ \varphi _j=\pm \frac{\chi }{2}, \end{aligned}$$

where the plus sign refers to $j=\textrm{I}$ and the minus sign to $j=\textrm{II}$. The absolute value diverges at phase shifter positions $\chi =\pm \pi$ due to the orthogonality between pre- and post-selection for this case. The phase $\varphi$ of the weak value is linear to the phase $\chi$ induced by the phase shifter. Real and imaginary part of the weak values are calculated as

6 $$\begin{aligned} \textrm{Re}\left\{ {\langle {{\hat{\Pi }}_j}\rangle }_\textrm{w}\right\} =\frac{1}{2}, \ \ \ \textrm{Im}\left\{ {\langle {{\hat{\Pi }}_j}\rangle }_\textrm{w}\right\} =\pm \frac{1}{2}\tan (\frac{\chi }{2}), \end{aligned}$$

with the same convention for the ± sign as above.

Consider the time-dependent oscillating external magnetic field of a radio-frequency (RF) spin-rotator coil

7 $$\begin{aligned} \textbf{B}(t)=\big (B_1 \cos (2\pi f t+\delta ),0, B_0\big )^\textrm{T}, \end{aligned}$$

with time *t*, the frequency *f* in resonance to the field strength $B_0$^36^ and the phase offset $\delta$ of the magnetic field in the coil at $t=0$. A neutron’s spin is manipulated by this external magnetic field which is described in the rotating wave approximation^37^ by the operator^36^

8 $$\begin{aligned} {\hat{U}}_\textrm{RF}(t, \alpha , f, \delta )=\begin{pmatrix} \cos \frac{\alpha }{2} & \textrm{i}\textrm{e}^{\textrm{i}(2\pi f t+\delta )}\sin \frac{\alpha }{2} \\ \textrm{i}\textrm{e}^{-\textrm{i}(2\pi f t+\delta )}\sin \frac{\alpha }{2} & \cos \frac{\alpha }{2} \end{pmatrix}= \begin{pmatrix} 1 & \textrm{i}\textrm{e}^{\textrm{i}(2\pi f t+\delta )}\frac{\alpha }{2} \\ \textrm{i}\textrm{e}^{-\textrm{i}(2\pi f t+\delta )}\frac{\alpha }{2} & 1 \end{pmatrix} + \mathcal {O}(\alpha ^2), \end{aligned}$$

in the *z* spin basis with the spin rotation angle $\alpha$. Each spin-flipped component is phase shifted by the phase $\delta$ of the magnetic field. The last step assumes the limit of weak interaction strengths, i.e., small rotation angles $\alpha \ll 1$.

The spin flip in an RF coil is coupled to an energy shift $\Delta E=h f$, with the Planck constant *h*, which manifests itself in a time-dependent relative phase $\pm 2\pi f t$ compared to the initial spin state. The interaction between the neutron and the magnetic field can be regarded in second quantisation of the magnetic field as a photon exchange^38,39^ with conserved energy and angular momentum. A neutron has a spin quantum number $S=1/2$. The spin flip of a neutron, described by a change $\pm \hbar =\pm h/(2\pi )$ in spin angular momentum, is compensated by the absorption or emission of a photon with the same angular momentum.

From now on, one RF spin-rotator is considered in each path $j\in \{\textrm{I}, \textrm{II}\}$ of the interferometer. We will distinguish the different frequencies of the coils $f_j$ and their different phase offsets $\delta _j$. We will use spin rotation angles $\alpha _\textrm{I}=\alpha _\textrm{II}=\alpha$. The path operators $\hat{\Pi }_j$ will indicate in which path an operation is conducted. In the ideal case, the intensity *I* after post-selection results as (detailed calculation in Supplementary Material)

9 $$\begin{aligned} \begin{aligned} I_\textrm{ideal}(t)=&\left| {\langle {\textrm{f}|\left( {\hat{U}}_\textrm{RF}(t,\alpha , f_\textrm{I}, \delta _\textrm{I})\hat{\Pi }_\textrm{I}+{\hat{U}}_\textrm{RF}(t,\alpha , f_\textrm{II}, \delta _\textrm{II})\hat{\Pi }_\textrm{II}\right) |\textrm{i}}\rangle }\right| ^2\\ =&\left| {\langle {\textrm{f}|\textrm{i}}\rangle }\right| ^2 \bigg [1 -\alpha \textrm{Im}\left\{ {\langle {{\hat{\Pi }}_\textrm{I}}\rangle }_\textrm{w} \textrm{e}^{-\textrm{i}(2\pi f_\textrm{I} t+\delta _\textrm{I})} +{\langle {{\hat{\Pi }}_\textrm{II}}\rangle }_\textrm{w} \textrm{e}^{-\textrm{i}(2\pi f_\textrm{II} t+\delta _\textrm{II})} \right\} + \mathcal {O}(\alpha ^2)\bigg ]\\ =&\left| {\langle {\textrm{f}|\textrm{i}}\rangle }\right| ^2 \bigg (1 -\alpha \bigg [ A_\textrm{I}\sin (\varphi _\textrm{I}-2\pi f_\textrm{I} t-\delta _\textrm{I}) +A_\textrm{II}\sin (\varphi _\textrm{II}-2\pi f_\textrm{II} t-\delta _\textrm{II}) \bigg ] + \mathcal {O}(\alpha ^2) \bigg ) , \end{aligned} \end{aligned}$$

where Eq. (4) is inserted for the last step. In the linear approximation for $\alpha \ll 1$ and with $(f_\textrm{I}-f_\textrm{II})/(f_\textrm{I}+f_\textrm{II})\ll 1$, this constitutes a temporal beating of the intensity. With a double-sine fit function for the time-resolved measurements, the absolute values $A_j$ will be directly extracted from the amplitudes of the intensity oscillations and the phases $\varphi _j$ of the weak values from the phases of the intensity oscillations (see “Extraction of weak values”). Real and imaginary components of the weak value are equivalent but secondary measures.

### Setup

**Fig. 1:**
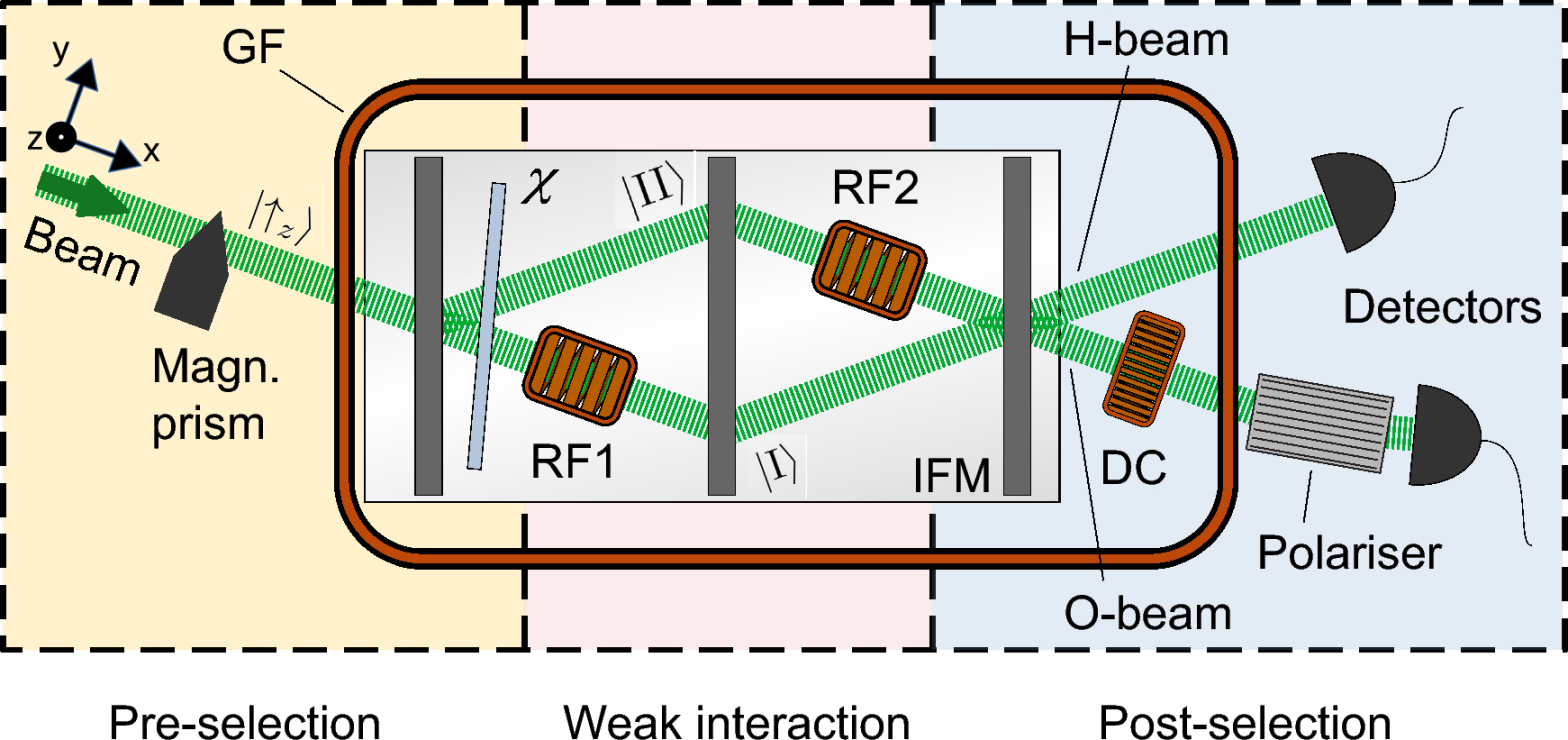
Setup of the neutron interferometer experiment. A monochromatised neutron beam is polarised in +*z*-direction by a magnetic prism. The interferometer crystal (IFM) splits the beam into the paths I and II. The sub-beams are recombined and the neutrons in the outgoing O-beam are detected with time resolution of 1 $\upmu$s. A guide field (GF) coil surrounds the experiment between prism and the polariser in the O-beam. The experiment consists of three stages: first, the pre-selection or preparation stage (light yellow) where the phase shifter determines the phase $\chi$, cf. Eq. (1). Second, the weak interaction stage (light red) where the spin is rotated by a rotation angle $\alpha =\pi /9$ in both radio-frequency (RF) spin manipulation coils RF1 and RF2. The coils are operated at frequencies $f_\textrm{I}=62.5$ kHz and $f_\textrm{II}=55.5$ kHz. Finally, the analysis or post-selection (light blue), cf. Eq. (2). At the recombination of the sub-beams, the incoming state is projected onto a specific phase relation between the sub-beams in the O-beam. There, the direct-current (DC) coil rotates the polarisation vector by $\pi /2$ around the *x*-direction. The polariser projects onto the +z spin state. The combination of DC coil and polariser acts as a spin analysis in +x-direction.

The experiment was conducted at the neutron interferometer station S18 at the Institut Laue-Langevin (ILL). The setup is depicted in Fig. 1. A neutron beam is monochromatised to a wavelength $$\lambda =1.92$$ Å, $$\delta \lambda /\lambda \approx 0.02$$, and polarised with a degree of polarisation $$P>0.99$$ in the vertical +*z*-direction, which defines the quantisation axis. The beam is split by the first plate of the single-crystal silicon neutron interferometer into two paths, denoted as $$j\in \{\textrm{I, II}\}$$. In both paths of the interferometer, a phase shifter is placed which is rotated to align the relative phase $$\chi$$ between the paths. After recombination of the two paths at the third plate of the interferometer crystal, two exiting beams emerge: the H-beam detected in diffracted direction and the O-beam detected in incident direction. In the present experiment, the O-beam exhibits a maximum count rate $N_\mathrm{max}$ of the order of $$\approx 10\, \mathrm{counts}/$$s and an experimental contrast $$C_\textrm{exp}=0.64\pm 0.03$$. As the single counting events cannot be correlated, Poisson statistics apply and any count rate $N$ is attributed with a statistical error $\sqrt{N}$. The intensity $I=N/N_\mathrm{max}$ in the O-beam with the neutron optical elements described so far is given as^30^

10 $$\begin{aligned} I_\textrm{O}=\frac{1}{2}(1+C_\textrm{exp}\cos \chi ). \end{aligned}$$

In the interferometer, an RF spin manipulating coil is operated in each path which realises the weak interactions of two path weak measurements simultaneously. The axes of the coils coincide with the local beam directions. Alternating currents are applied with radio-frequencies $f_\textrm{I}=62.5$ kHz in path I and $f_\textrm{II}=55.5$ kHz in path II. The amplitudes are adjusted to realise spin rotation angles $\alpha =\pi /9\pm \pi /720\hat{=}(20\pm 0.5)$ degree^40^ with a spin manipulation efficiency $\epsilon _\textrm{exp}=0.92\pm 0.02$. The vertical fields $B_{0,j}$ in the RF coils are generated by a large Helmholtz coil, which surrounds the interferometer and the O-beam, and a small Helmholtz coil at each RF coil for fine tuning of the resonance^41^. In the O-beam, a direct-current (DC) coil is positioned which applies a $\pi /2$ rotation of the polarisation vector around the local *x*-direction. Further downstream, a polariser transmits the spin-up component. The sequence of DC coil and polariser allows the $+x$ spin analysis assumed in Eq. (2). With the $+x$ spin analysis, the initial spin up component and spin down component produced by the weak interaction interfere^42,43^.

The RF signals are reset every 304 $\upmu$s by the trigger of a function generator. In the O-beam, a mean count rate of up to 2 neutron counts per interval is expected. The detector in the O-beam registers the arrival time of the neutrons with a time-resolution of $1\,\upmu$s. Each binning time corresponds to a neutron which interacted with a magnetic field induced by the RF coils of a specific phase. To improve the counting statistics, the time-dependent measurements are repeated for 1 h. Due to the low intensity given by Eq. (10) in the vicinity of $\chi =\pm \pi$, the measurements are extended to 2 h at the closest points of $\chi =\pm 35/36\ \pi$. The sum of all time-dependent measurements at the same phase shifter position $\chi$ is called a time spectrum.

The thermal control and water cooling^41,44^ system stabilises the temperature sensitive interferometer to phase drifts $\lesssim$1 degree/h. Additionally, the distance between the RF coils is maximised by installing them in different sections of the interferometer as depicted in Fig. 1 to reduce the mutual influences between the weak interactions.

### Experimental results

**Fig. 2:**
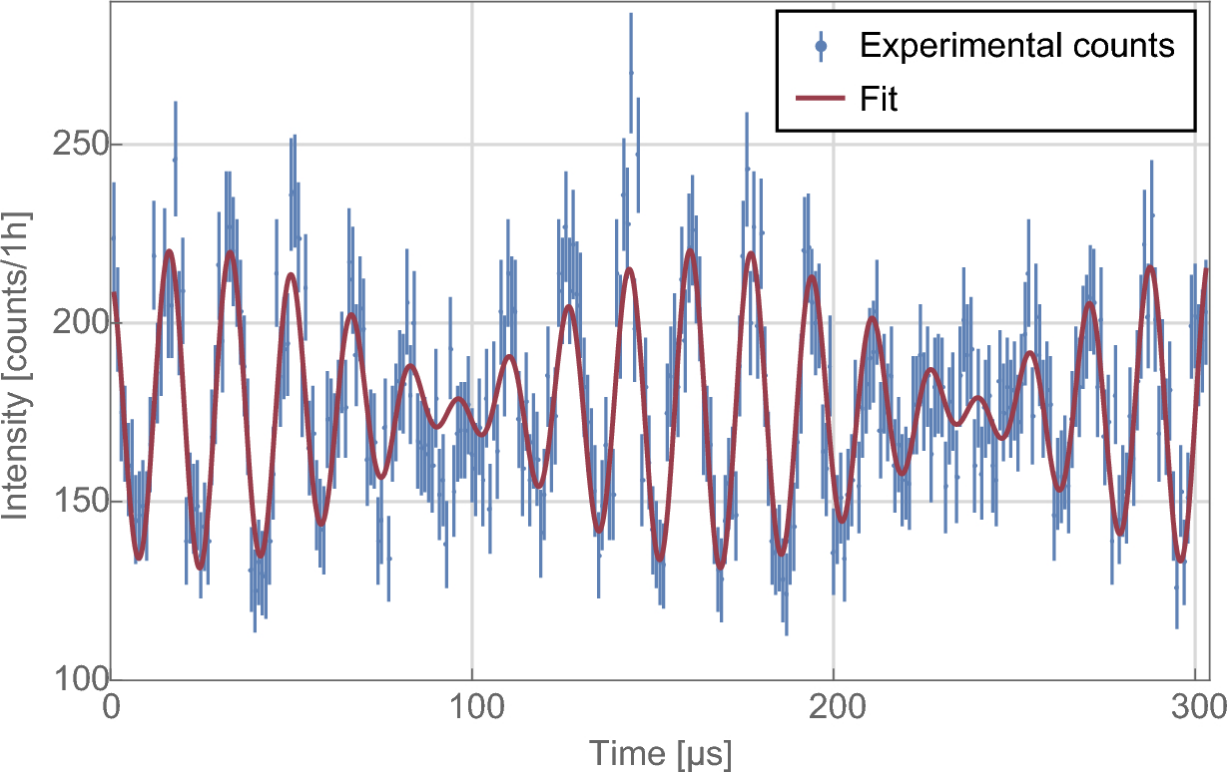
Example of a time spectrum of the count rate at relative phase $\chi =0$. Data points in blue. Error bars indicate one statistical standard deviation. The time axis is with regards to the trigger for the reset of the signals to both radio-frequency coils every 304 $\upmu$s (see “Setup”). Time-resolved measurements are repeated over 1 h and summed up to the depicted time spectrum. The count rate is beating in time following the theoretical prediction of Eq. (9). Solid red line is a fit according to Eq. (11).

The time-resolved count rates in the O-beam were recorded for several phase shifter settings $\chi$. An example of such a recording is depicted in Fig. 2. A beating modulation of the count rate is observed as predicted per Eq. (9). The intensities are converted into intensities and fitted with a corresponding double-sine fit function,

11 $$\begin{aligned} I_\textrm{fit}(t)=I_{0, \textrm{fit}}+D_\textrm{I}\sin (2\pi f_\textrm{I}t+\phi _\textrm{I})+D_\textrm{II}\sin (2\pi f_\textrm{II}t+\phi _\textrm{II}) \end{aligned}$$

with the mean intensity $I_{0, \textrm{fit}}$ and oscillations with amplitudes $D_j$ and phases $\phi _j$, $j\in \{\textrm{I, II}\}$.

The amplitudes $D_j$ and phases $\phi _j$ of the oscillations naturally offer the direct extraction of the complex weak values ${\langle {{\hat{\Pi }}_j}\rangle }_\textrm{w}=A_j\textrm{e}^{\textrm{i}\varphi }$ in polar coordinates. The experimentally retrieved values, indexed with “exp”, of absolute value $A_{j,\,\textrm{exp}}$ and phase $\varphi _{j,\,\textrm{exp}}$ of the weak value are given for the linear approximation in $\alpha$ as (see “Extraction of weak values” for derivation)

12 $$\begin{aligned} A_{j,\,\textrm{exp}}=\frac{D_j}{\alpha I_{0, \textrm{fit}}},\ \ \ \ \varphi _{j,\,\textrm{exp}}=\delta _{j,\,\textrm{exp}}-\phi _j. \end{aligned}$$

**Fig. 3:**
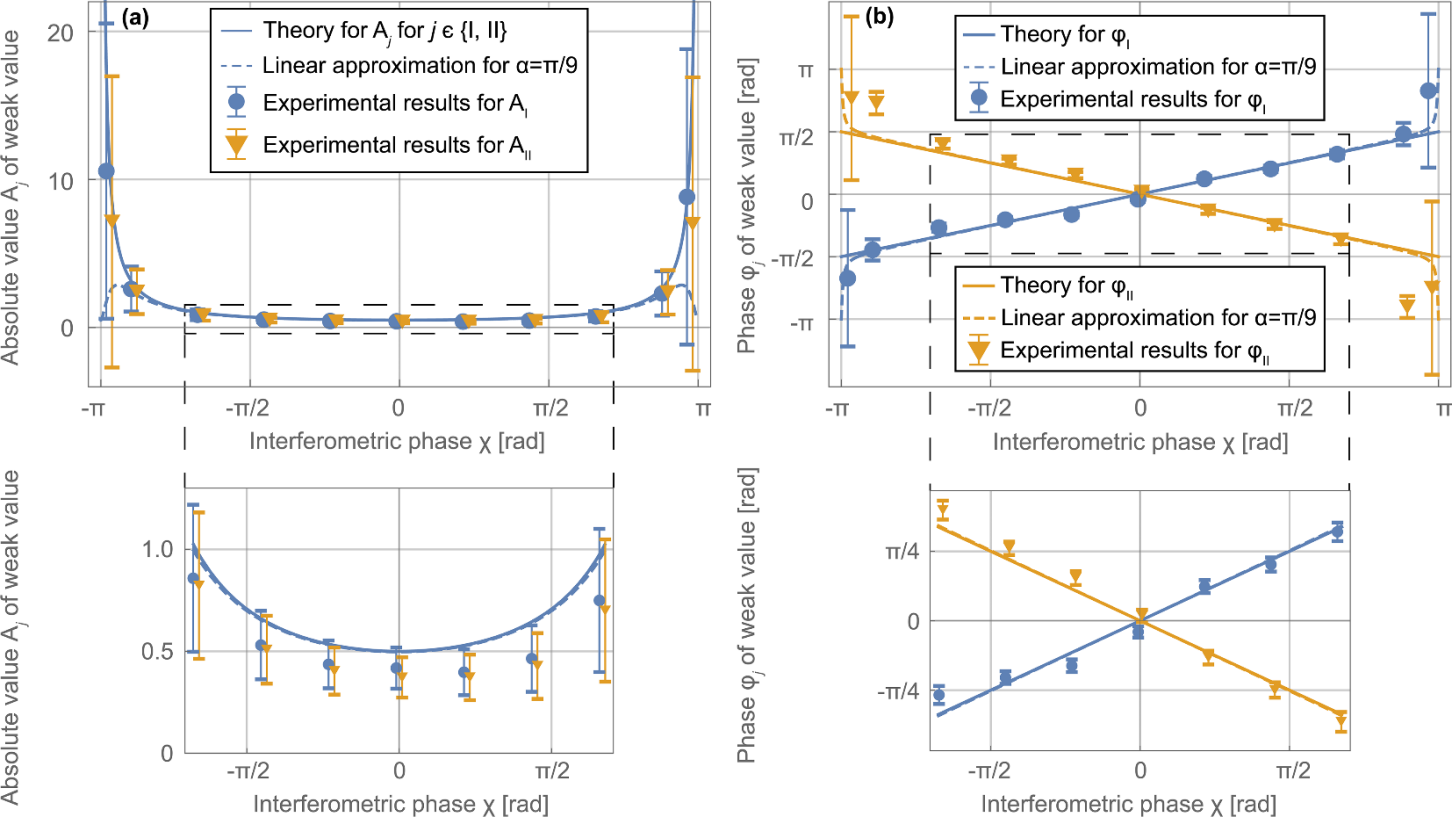
The complex weak values in polar coordinates ${\langle {{\hat{\Pi }}_j}\rangle }_\textrm{w}=A_j\,\textrm{e}^{\textrm{i}\varphi _j}$. They are given through (**a**) the absolute value $A_j$ and (**b**) the phase $\varphi _j$ dependent on the interferometric phase shift $\chi$ with theoretical predictions. In (**a**) and (b), the dashed frame is magnified on the bottom. Error bars indicate one standard deviation and include the systematic errors. Solid curves are the theoretical predictions in the limit of the spin rotation angle $\alpha \ll 1$. Dashed curves indicate the expected behaviour of the linear data analysis used with the applied finite spin rotation angle $\alpha =\pi /9$. Where the solid and dashed lines separate, the linear approximation in $\alpha$ of Eq. (9) does not hold anymore.

**Fig. 4:**
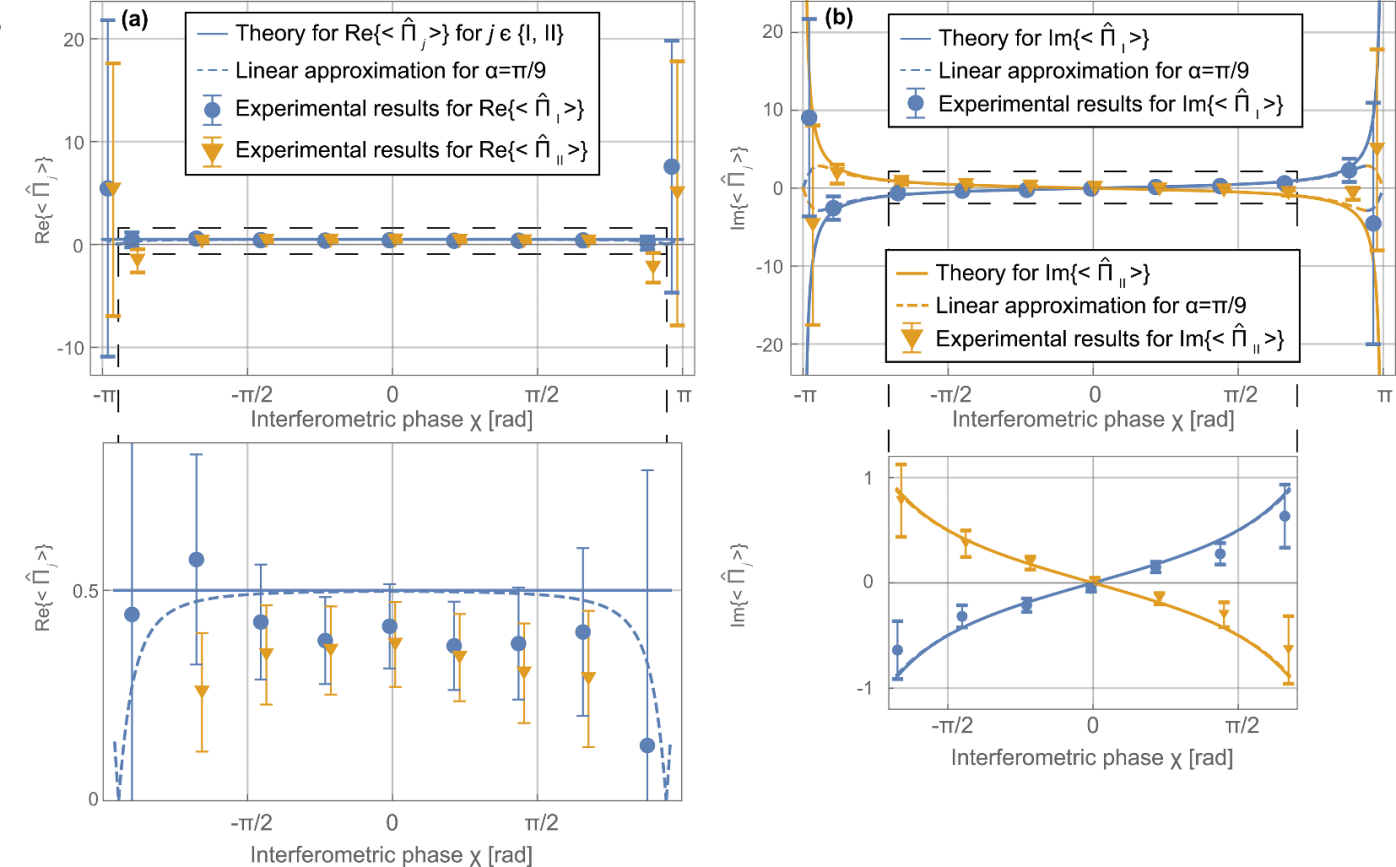
The complex weak values ${\langle {{\hat{\Pi }}_j}\rangle }_\textrm{w}$ given through (**a**) the real and (**b**) the imaginary part dependent on the interferometric phase shift $\chi$ with theoretical predictions. In (**a**) and (**b**), the dashed frame is magnified on the bottom. Error bars indicate one standard deviation and include the systematic errors. Solid curves are the theoretical prediction in the limit of the spin rotation angle $\alpha \ll 1$. Dashed curves indicate the expected behaviour of the linear data analysis used with the applied finite spin rotation angle $\alpha =\pi /9$. Where the solid and dashed lines separate, the linear approximation in $\alpha$ of Eq. (9) does not hold anymore.

After the correction procedure described in “Data Correction”, these absolute values and phases of the path weak values are plotted in Fig. 3 dependent on the interferometer phase $\chi$ between the two paths. The absolute value diverges at interferometer phases $\chi =\pm \pi$ as predicted by Eq. (5). The phases of the weak values are linear in $\chi$ as predicted by Eq. (5). This directly represents the phase of the intensity modulation in time. By changing the basis to real and imaginary coordinates, the data in Fig. 4 arise. Theory predicts that the real part is constant for all $\chi$. The imaginary parts are anti-symmetric functions which also diverge for $\chi =\pm \pi$. Note that the polar coordinates are extracted directly from fitting a double-sine curve as in Eqs. (9) and (11), while the real and imaginary components are equivalent but secondary measures. It is clearly seen that the extracted data agree with the theoretical predictions. Simultaneous weak-measurements are accomplished.

## Discussion

A simultaneous one-shot measurement of 2 weak values is conducted by implementing sinusoidal oscillations of the magnetic fields with different frequencies in each path. Time-resolved measurements of the intensity are used to directly extract the weak values in polar coordinates from the fitting parameters of a double-sine fit. The absolute values and phases of the weak values are furthermore converted to the real and imaginary components. For the present experiment, this indicates that the absolute value and phase are the measures with direct physical significance rather than the secondary real and imaginary components.

While it is not anymore necessary in the presented procedure to turn the devices for the weak interactions on and off, a time-dependent measurement is necessary to retrieve multiple weak values. This can be regarded as trading in one experimental requirement with another. However, the presented results are extracted with a single ensemble at each phase shifter setting $\chi$. The simultaneous validity of the 2 weak values is therefore guaranteed while preserving the studied interference effect. We want to emphasise that the simultaneously extracted weak values agree with the predictions for single weak values.

We will now discuss the connection between the neutron optical elements and the measured intensity. The mean intensity $\bar{I}_\textrm{O}$ in the O-beam of Fig. 2 for $\alpha \ll 1$ is given by $I_\mathrm{O}$ of Eq. (10), multiplied by another factor of 1/2 due to the spin analysis in +*x*-direction of the initial $+z$ spin state. The time-dependence of the weak interaction makes the intensity time-dependent. The beating intensity modulation of Fig. 2 originates in the sinusoidal RF signal. Let us first consider only a single applied RF signal. The time of entry into the RF field determines the relative phase at the end of the coil between the flipped spin down component and the initial spin up component. The relative phase between spin up and down component regulates the $+x$-component of the spin state and in effect the transmission probability at the spin analysis. This relative phase is time-dependent after exiting the coil, due to the energy shift. Therefore, the polarisation vector rotates^36^. With the spin analysis in $+x$-direction, an oscillating intensity is observed with one coil. With two coils operated at different frequencies, two such oscillations can be directly added in the limit $\alpha \ll 1$ such that a beating emerges if $(f_\textrm{I}-f_\textrm{II})/(f_\textrm{I}+f_\textrm{II})\ll 1$. The weak values are directly given by the amplitude and phase of the oscillations via Eq. (9). For a fixed $\alpha$, the amplitude is connected to the probability amplitude in the respective path and in turn to the path operator. The phase of the magnetic field determines the phase of the detected intensity modulation. Therefore, the magnetic fields of the RF coils encode the weak values into the intensity. (The spin rotation angle $\alpha$ scales the amplitude of the time-dependent signal with the absolute value of the weak value.)

Wave and particle are complementary properties^45^. The visibility of interference fringes and distinguishability between paths are related through a trade-off^46–48^. A spin flip of probability 1 in one path of an interferometer makes the paths distinguishable through a spin analysis while the visibility vanishes due to the orthogonality between the spin states. A so-called quantum eraser makes the paths indistinguishable again which can be implemented by projecting the orthogonal states onto a different basis. This was done in neutron interferometer experiments^38^ and in versions with entangled photons^49–51^.

In the present experiment, the spin analysis in +x-direction resembles the quantum eraser. Some minimal path marking is memorised in the spin system given through the spin rotation angle $\alpha$. As the same rotation angle $\alpha$ from the initial +z spin state is applied in both paths, the paths cannot be distinguished by projecting on a z spin state. However, the two polarisation vectors are rotating with different frequencies in an x–y plane of the Bloch sphere. The x–y spin components are periodically in parallel and anti-parallel. For the anti-parallel orientation, the corresponding spin states are orthogonal. If all parameters of the experiment were determined, the detection of a neutron at specific moments would infer different probabilities for either path taken. In contrast to a quantum eraser, some minimal path distinguishability could be inferred with the combination of +x spin analysis and time-resolved detection. Since both flipped components are involved for this argument, the reduced visibility emerges in the intensity with the second order in $\alpha$. Due to the small size of $\alpha$, the present experiment is adequately described by the linear dependence on $\alpha$. The minimal distinguishability between the paths does not lower the visibility noticeably.

The extracted weak values of Figs. 3 and 4 conform mostly with the theoretical prediction. When the interferometer phase $\chi$ approaches $\pm \pi$, the absolute value and the imaginary part of the weak values diverge according to Eqs. (5) and (6). Consequently, the error bars drastically increase at these phase shifter positions in both the polar and Cartesian representation.

The absolute values of Fig. 3 are systematically too small. This is caused in the time-resolved measurements (cf. Fig. 2) by amplitudes of the oscillations which are lower than predicted. We attribute this to a systematically too small spin rotation angle $\alpha$ which we estimate to be too low by a factor of $\approx 0.8$. For $\alpha =\pi /9\,\hat{=}\,20$ degree, this is an order of magnitude more than the uncertainty of $\pm 0.5$ degree as estimated in “Setup”. The phase $\varphi _\textrm{II}$ also exhibits a systematic deviation from theory. The slope of the data points for $\varphi _\textrm{II}$ is steeper than expected. We attribute this to the reset of the field every 304 $\upmu$s: while this is a multiple of the oscillation period for $f_\textrm{I}$, the oscillation with $f_\textrm{II}$ is cut off at the reset. The magnetic field inside the coil and its driving stimulus are then off-phase which can explain some phase deviations.

The data extraction uses the double-sine fit which assumes small rotation angles $\alpha \ll 1$. However, we apply a finite rotation angle $\alpha =\pi /9$. For finite rotation angles, deviations from the linear approximation in $\alpha$ of the extracted values due to higher order terms are expected. These deviations are depicted in the graphs as dashed lines. (They assume single weak value extraction and omit the cross-term between the spin down components of both paths. Equation (16) in “Data Correction” explicitly states this cross-term $\sim \sin ^2\left( \alpha /2\right)$ just before the approximation.) The extracted data comply with both the first order approximation and the expected deviations due to the size of the error bars. Therefore, the chosen spin rotation angle is suitable for the chosen combination of setup and measurement time and the linear approximation in $\alpha$ is reasonable.

The systematic effects described for the polar coordinates are also visible in Fig. 4 in the equivalent terms of real and imaginary part of the weak values. For large interferometer phases $\chi$, the errors are increased. The systematically reduced absolute values compared to prediction translate in the systematically reduced real and imaginary parts. The real part is usually interpreted as the best estimate for the path operators^10,11^. One can interpret the estimates in terms of detection probabilities. For our symmetric beam splitter, a real part of 1/2 is predicted. In the comparison of the two sets of real parts, the deviations between both sets are comparable with the error bars. This is consistent with both paths being equally probable and visibility of the interference fringes is maintained. The second and second-last real parts of the yellow data set $\textrm{Re}\{{\hat{\Pi }}_\textrm{II}\}$ are incompatible with the prediction. This is linked to the similarly incompatible phases $\varphi _\textrm{II}$ of the weak value in Fig. 3.

The absolute values of the imaginary part are also systematically too small. The phase inaccuracy is visible here, too. The imaginary parts are often interpreted as intrinsic measurement disturbance^11,12^. The divergences of the imaginary parts at $\chi =\pm \pi$ are connected to the vanishing intensity at the detector such that no information can be gained. The change in sign for the left and right-most points is consistent with the theory prediction for the finite angle of $\alpha$. The sum of the imaginary parts is expected to be zero because the sum of the path weak values is supposed to be 1$\in \mathbb {R}$.

Although the presented weak values are valid for the same ensemble, they do not necessarily characterise individual neutrons. Yet, a recent experimental report^52^ characterises individual neutrons with weak values.

The extraction method through time-resolved measurements can be extended to an arbitrary number of paths with as many different frequencies, since the number of energy levels used is, in principle, not limited. In case of neutron interferometer experiments, three simultaneous weak path measurements can be the next target. In comparison, implementing spatial shifts as a path marking with a detector with a 2-dimensional spatial resolution is limited to the extraction of 2 weak values. Further experiments will involve simultaneous measurements of weak values in the case of a magnetic field along a single axis^53^ and asymmetric beam splitters.

## Conclusion

Simultaneous measurements of two path weak values in neutron interferometry are presented. The path degree of freedom of the neutron is coupled to the energy degree of freedom through weak interactions with oscillating magnetic fields. By use of two different frequencies, two different energy eigenstates are occupied which act as two probe states. Since the choice of the frequencies has no limitation, the potential number of probe states and extracted path weak values in the experiment is unlimited. By recording the time-dependent intensity, two path weak values are simultaneously extracted as full complex numbers at each phase shifter position. This simplified measurement protocol guarantees the validity of multiple weak values for the same ensemble.

In contrast to strong measurements, e.g., with beam blockers or $\pi$ spin rotations in the interferometer arms, weak measurements preserve the investigated interference effect. In the present experiment, the principal physical significance of the weak values lies in their polar coordinates of absolute value and phase which directly characterise the amplitude and phase of the observed intensity modulation. Further experiments will involve the extension to simultaneous measurements of three or more weak values and the use of asymmetric beam splitters.

## Methods

### Extraction of weak values

To extract the weak values from the measured intensities, we equate the linear approximation in $\alpha$ of Eq. (9) for the ideal case from theory with the fit function of Eq. (11). Quantities with index “exp” refer to the experimentally retrieved values in contrast to the theoretical prediction:

13 $$\begin{aligned} \begin{aligned} I_\textrm{ideal}(t)&=I_\textrm{fit}(t)\\ \Rightarrow \left| {\langle {\textrm{f}|\textrm{i}}\rangle }\right| ^2 \bigg (1-\alpha \bigg [ A_\textrm{I, exp}\sin (\varphi _\textrm{I, exp}-2\pi f_\textrm{I} t-\delta _\textrm{I, exp})&+A_\textrm{II, exp}\sin (\varphi _\textrm{II, exp}-2\pi f_\textrm{II} t-\delta _\textrm{II, exp}) \bigg ] \bigg )\\&= I_{0, \textrm{fit}}+D_\textrm{I}\sin (2\pi f_\textrm{I}t+\phi _\textrm{I})+D_\textrm{II}\sin (2\pi f_\textrm{II}t+\phi _\textrm{II}) . \end{aligned} \end{aligned}$$

By comparing the coefficients to the left and right, we obtain

14 $$\begin{aligned} \begin{aligned} \left| {\langle {\textrm{f}|\textrm{i}}\rangle }\right| ^2=&I_{0, \textrm{fit}}, \\ \left| {\langle {\textrm{f}|\textrm{i}}\rangle }\right| ^2 \alpha A_\textrm{I, exp}\sin (2\pi f_\textrm{I} t+\delta _\textrm{I, exp}-\varphi _\textrm{I, exp})=&D_\textrm{I}\sin (2\pi f_\textrm{I}t+\phi _\textrm{I}), \\ \left| {\langle {\textrm{f}|\textrm{i}}\rangle }\right| ^2 \alpha A_\textrm{II, exp}\sin (2\pi f_\textrm{II} t+\delta _\textrm{II, exp}-\varphi _\textrm{II, exp})=&D_\textrm{II}\sin (2\pi f_\textrm{II}t+\phi _\textrm{II}), \\ \Rightarrow A_\textrm{I, exp}=\frac{D_{\textrm{I}}}{\alpha I_{0, \textrm{fit}}},\ \ \ \ &A_\textrm{II, exp}=\frac{D_{\textrm{II}}}{\alpha I_{0, \textrm{fit}}}, \\ \varphi _\textrm{I, exp}=\delta _\textrm{I, exp}-\phi _\textrm{I},\ \ \ \ &\varphi _\textrm{II, exp}=\delta _\textrm{II, exp}-\phi _\textrm{II} . \end{aligned} \end{aligned}$$

The absolute phase between the paths of the interferometer is unknown and only the relative phase $\chi$ is controlled. Therefore, the phase offsets $\delta _j$ are also undetermined albeit predictable. However, according to Eq. (5), the phases $\varphi _j$ of the weak value are anti-symmetric in $\chi$ and their mean is zero. So we chose the phase offsets $\delta _{j, \textrm{exp}}$ such that both means of the phases $\varphi _j$ of the time-dependent oscillation are zero, ${\bar{\varphi }}_j=0$. The data correction is described in the next section.

### Data correction

In the ideal case, the weak values are extracted in polar coordinates through Eq. (14). The dominant experimental imperfections are given by the interferometer contrast *C* and the coil efficiency $\epsilon$. The experimentally retrieved parameters are $C_\textrm{exp}=0.64\pm 0.03$ and $\epsilon _\textrm{exp}=0.92\pm 0.02$. The predicted experimental intensity $I_\textrm{pred}$ dependent on the adjustable parameters $\alpha _j$ and $\chi$ is derived as

15 $$\begin{aligned} \begin{aligned} I_\textrm{p}&_\textrm{red}(t)\\ =&\left| {\langle {\textrm{f}|\left( {\hat{U}}_\textrm{RF}(t, \alpha _\textrm{I}, f_\textrm{I}, \delta _\textrm{I}){\hat{\Pi }}_\textrm{I}+{\hat{U}}_\textrm{RF}(t, \alpha _\textrm{II}, f_\textrm{II}, \delta _\textrm{II}){\hat{\Pi }}_\textrm{II}\right) |\textrm{i}}\rangle }\right| ^2\\ =&\Bigg |\frac{1}{\sqrt{2}}\big ({\langle {\textrm{I}}|}+{\langle {\textrm{II}}|}\big ){\langle {\uparrow _x}|} \frac{1}{\sqrt{2}} \bigg [ {|{\textrm{I}}\rangle } \left( \cos \left( \frac{\alpha _\textrm{I}}{2}\right) {|{\uparrow _z}\rangle }+\textrm{i}\sin \left( \frac{\alpha _\textrm{I}}{2}\right) \textrm{e}^{-\textrm{i}(2\pi f_\textrm{I} t+\delta _\textrm{I})}{|{\downarrow _z}\rangle }\right) \\&\hspace{4.5cm} +\textrm{e}^{-\textrm{i}\chi }{|{\textrm{II}}\rangle } \left( \cos \left( \frac{\alpha _\textrm{II}}{2}\right) {|{\uparrow _z}\rangle } +\textrm{i}\sin \left( \frac{\alpha _\textrm{II}}{2}\right) \textrm{e}^{-\textrm{i}(2\pi f_\textrm{II} t+\delta _\textrm{II})} {|{\downarrow _z}\rangle }\right) \bigg ] \Bigg |^2\\ =&\bigg |\frac{1}{\sqrt{2}}\big ({\langle {\textrm{I}}|}+{\langle {\textrm{II}}|}\big )\frac{1}{\sqrt{2}}\left( {\langle {\uparrow _z}|}+{\langle {\downarrow _z}|}\right) \\ \times&\frac{1}{\sqrt{2}}\bigg [ {|{\textrm{I}}\rangle }\left( \cos \left( \frac{\alpha _\textrm{I}}{2}\right) {|{\uparrow _z}\rangle }+\textrm{i}\sin \left( \frac{\alpha _\textrm{I}}{2}\right) \textrm{e}^{-\textrm{i}(2\pi f_\textrm{I} t+\delta _\textrm{I})}{|{\downarrow _z}\rangle }\right) +\textrm{e}^{-\textrm{i}\chi }{|{\textrm{II}}\rangle }\left( \cos \left( \frac{\alpha _\textrm{II}}{2}\right) {|{\uparrow _z}\rangle } +\textrm{i}\sin \left( \frac{\alpha _\textrm{II}}{2}\right) \textrm{e}^{-\textrm{i}(2\pi f_\textrm{II} t+\delta _\textrm{II})} {|{\downarrow _z}\rangle }\right) \bigg ] \bigg |^2\\ =&\left| \frac{1}{2\sqrt{2}} \left[ \left( \cos \left( \frac{\alpha _\textrm{I}}{2}\right) +\textrm{i}\sin \left( \frac{\alpha _\textrm{I}}{2}\right) \textrm{e}^{-\textrm{i}(2\pi f_\textrm{I} t+ \delta _\textrm{I})}\right) +\textrm{e}^{-\textrm{i}\chi }\left( \cos \left( \frac{\alpha _\textrm{II}}{2}\right) +\textrm{i}\sin \left( \frac{\alpha _\textrm{II}}{2}\right) \textrm{e}^{-\textrm{i} (2\pi f_\textrm{II} t+\delta _\textrm{II})} \right) \right] \right| ^2\\ =&\frac{1}{8}\bigg [ \cos ^2\left( \frac{\alpha _\textrm{I}}{2}\right) + \sin ^2\left( \frac{\alpha _\textrm{I}}{2}\right) +\textrm{e}^{\textrm{i}\chi }\left( \cos \left( \frac{\alpha _\textrm{II}}{2}\right) -\textrm{i}\sin \left( \frac{\alpha _\textrm{II}}{2}\right) \textrm{e}^{+\textrm{i}(2\pi f_\textrm{II} t+\delta _\textrm{II})} \right) \textrm{e}^{-\textrm{i}\chi }\left( \cos \left( \frac{\alpha _\textrm{II}}{2}\right) +\textrm{i}\sin \left( \frac{\alpha _\textrm{II}}{2}\right) \textrm{e}^{-\textrm{i}(2\pi f_\textrm{II} t+\delta _\textrm{II})} \right) \\&-\textrm{i}\sin \left( \frac{\alpha _\textrm{I}}{2}\right) \textrm{e}^{+\textrm{i}(2\pi f_\textrm{I} t+\delta _\textrm{I})}\cos \left( \frac{\alpha _\textrm{I}}{2}\right) + \cos \left( \frac{\alpha _\textrm{I}}{2}\right) \textrm{i}\sin \left( \frac{\alpha _\textrm{I}}{2}\right) \textrm{e}^{-\textrm{i}(2\pi f_\textrm{I} t+\delta _\textrm{I})} \\&+ \cos \left( \frac{\alpha _\textrm{I}}{2}\right) \left[ \textrm{e}^{-\textrm{i}\chi }\left( \cos \left( \frac{\alpha _\textrm{II}}{2}\right) +\textrm{i}\sin \left( \frac{\alpha _\textrm{II}}{2}\right) \textrm{e}^{-\textrm{i}(2\pi f_\textrm{II} t+\delta _\textrm{II})} \right) + \textrm{e}^{\textrm{i}\chi }\left( \cos \left( \frac{\alpha _\textrm{II}}{2}\right) -\textrm{i}\sin \left( \frac{\alpha _\textrm{II}}{2}\right) \textrm{e}^{+\textrm{i}(2\pi f_\textrm{II} t+\delta _\textrm{II})} \right) \right] \\&+ \textrm{e}^{\textrm{i}\chi }\left( \cos \left( \frac{\alpha _\textrm{II}}{2}\right) -\textrm{i}\sin \left( \frac{\alpha _\textrm{II}}{2}\right) \textrm{e}^{+\textrm{i}(2\pi f_\textrm{II} t+\delta _\textrm{II})} \right) \textrm{i}\sin \left( \frac{\alpha _\textrm{I}}{2}\right) \textrm{e}^{-\textrm{i}(2\pi f_\textrm{I} t+\delta _\textrm{I})} \\&- \textrm{i}\sin \left( \frac{\alpha _\textrm{I}}{2}\right) \textrm{e}^{+\textrm{i}(2\pi f_\textrm{I} t+\delta _\textrm{I})}\textrm{e}^{-\textrm{i}\chi }\left( \cos \left( \frac{\alpha _\textrm{II}}{2}\right) +\textrm{i}\sin \left( \frac{\alpha _\textrm{II}}{2}\right) \textrm{e}^{-\textrm{i}(2\pi f_\textrm{II} t+\delta _\textrm{II})} \right) \bigg ] . \end{aligned} \end{aligned}$$

Here, we insert the experimental parameters of interferometer contrast *C* and the spin manipulation efficiency $\epsilon$ of the coils. The contrast is added in summands with factors attributed to both paths, which is equivalent to the dependence on both $\alpha _\textrm{I}$ and $\alpha _\textrm{II}$. From here on, we set $\alpha _\textrm{I}=\alpha _\textrm{II}=\alpha$. The spin manipulation efficiency is added in summands with factors attributed to different spin components. The latter is equivalent to adding the factor $\epsilon$ in summands with factors $\sin (\alpha /2)\cos (\alpha /2)$, where the cosine quantifies the component remaining in the initial +z spin state and the sine quantifies the component flipped into the -z spin state.

16 $$\begin{aligned} \begin{aligned} I_\textrm{p}&_\textrm{red}(t)\\ =&\frac{1}{8}\bigg [ 1+\cos ^2\left( \frac{\alpha }{2}\right) + \sin ^2\left( \frac{\alpha }{2}\right) -\epsilon \,\textrm{i}\sin \left( \frac{\alpha }{2}\right) \textrm{e}^{+\textrm{i}(2\pi f_\textrm{II} t+\delta _\textrm{II})} \cos \left( \frac{\alpha }{2}\right) +\epsilon \cos \left( \frac{\alpha }{2}\right) \textrm{i}\sin \left( \frac{\alpha }{2}\right) \textrm{e}^{-\textrm{i}(2\pi f_\textrm{II} t+\delta _\textrm{II})} \\&+2\epsilon \sin \left( 2\pi f_\textrm{I} t+\delta _\textrm{I}\right) \sin \left( \frac{\alpha }{2}\right) \cos \left( \frac{\alpha }{2}\right) \\&+2 C \cos \left( \chi \right) \cos \left( \frac{\alpha }{2}\right) \cos \left( \frac{\alpha }{2}\right) + 2 \epsilon C \sin \left( 2\pi f_\textrm{II} t+\delta _\textrm{II}+\chi \right) \cos \left( \frac{\alpha }{2}\right) \sin \left( \frac{\alpha }{2}\right) \\&+ 2 \epsilon C \sin \left( 2\pi f_\textrm{I} t+\delta _\textrm{I}-\chi \right) \sin \left( \frac{\alpha }{2}\right) \cos \left( \frac{\alpha }{2}\right) +2 C \cos \left( (2\pi f_\textrm{I}-2\pi f_\textrm{II})t+\delta _\textrm{I}-\delta _\textrm{II}-\chi \right) \sin ^2\left( \frac{\alpha }{2}\right) \bigg ] \\ =&\frac{1}{4}\bigg [ 1+ C \cos \left( \chi \right) \cos ^2\left( \frac{\alpha }{2}\right) \\&+\epsilon \bigg (\sin \left( 2\pi f_\textrm{I} t+\delta _\textrm{I}\right) \sin \left( \frac{\alpha }{2}\right) \cos \left( \frac{\alpha }{2}\right) + \sin \left( 2\pi f_\textrm{II} t+\delta _\textrm{II}\right) \sin \left( \frac{\alpha }{2}\right) \cos \left( \frac{\alpha }{2}\right) \\&+ C \sin \left( 2\pi f_\textrm{II} t+\delta _\textrm{II}+\chi \right) \cos \left( \frac{\alpha }{2}\right) \sin \left( \frac{\alpha }{2}\right) + C \sin \left( 2\pi f_\textrm{I} t+\delta _\textrm{I}-\chi \right) \sin \left( \frac{\alpha }{2}\right) \cos \left( \frac{\alpha }{2}\right) \bigg )\\&+ C \cos \left( (2\pi f_\textrm{I}-2\pi f_\textrm{II})t+\delta _\textrm{I}-\delta _\textrm{II}-\chi \right) \sin ^2\left( \frac{\alpha }{2}\right) \bigg ] \\ \approx&\frac{1}{4}\bigg [ 1+ C \cos \left( \chi \right) +\frac{\epsilon \alpha }{2} \bigg (\sin \left( 2\pi f_\textrm{I} t+\delta _\textrm{I}\right) + \sin \left( 2\pi f_\textrm{II} t+\delta _\textrm{II}\right) + C \big (\sin \left( 2\pi f_\textrm{I} t+\delta _\textrm{I}-\chi \right) \big ) +\sin \left( 2\pi f_\textrm{II} t+\delta _\textrm{II}+\chi \right) \bigg )\bigg ]\\ =:&I_0+I(t) \\ =&I_0+ \frac{\epsilon \alpha }{8}\bigg [ \sin \left( 2\pi f_\textrm{I} t+\delta _\textrm{I}\right) +\sin \left( 2\pi f_\textrm{II} t+\delta _\textrm{II}\right) \\&+ C \bigg (\sin \left( 2\pi f_\textrm{I} t+\delta _\textrm{I}\right) \cos \left( \chi \right) - \cos \left( 2\pi f_\textrm{I} t+\delta _\textrm{I}\right) \sin \left( \chi \right) + \sin \left( 2\pi f_\textrm{II} t+\delta _\textrm{II}\right) \cos \left( \chi \right) + \cos \left( 2\pi f_\textrm{II} t+\delta _\textrm{II}\right) \sin \left( \chi \right) \bigg )\bigg ]\\ =&I_0+ \frac{\epsilon \alpha }{8}\bigg [ \sin \left( 2\pi f_\textrm{I} t+\delta _\textrm{I}\right) \big (1-C\cos \left( \chi \right) \big ) +\cos \left( 2\pi f_\textrm{I} t+\delta _\textrm{I}\right) C\sin \left( \chi \right) \\&+\sin \left( 2\pi f_\textrm{II} t+\delta _\textrm{II}\right) \big (1+C\cos \left( \chi \right) \big ) +\cos \left( 2\pi f_\textrm{II} t+\delta _\textrm{II}\right) C\sin \left( \chi \right) \bigg ], \\ \end{aligned} \end{aligned}$$

with $I_0=\left[ 1+ C \cos \left( \chi \right) \right] /4$. There is a sine and a cosine summand for each applied frequency. We combine each pair by defining new amplitudes $R_j$ and phases $\beta _j$.

17 $$\begin{aligned} \begin{aligned} I_\textrm{exp}(t)=:&I_0 - \alpha \bigg ( R_\textrm{I}\sin \left( \beta _\textrm{I}-2\pi f_\textrm{I} t-\delta _\textrm{I}\right) + R_\textrm{II}\sin \left( \beta _\textrm{II}-2\pi f_\textrm{II} t-\delta _\textrm{II}\right) \bigg ) \\ =&I_0 - \alpha \bigg ( R_\textrm{I}\big (\sin \left( 2\pi f_\textrm{I} t+\delta _\textrm{I}\right) \cos \left( \beta _\textrm{I}\right) +\cos \left( 2\pi f_\textrm{I} t+\delta _\textrm{I}\right) \sin \left( \beta _\textrm{I}\right) \big )\\&+ R_\textrm{II}\big (\sin \left( 2\pi f_\textrm{II} t+\delta _\textrm{II}\right) \cos \left( \beta _\textrm{II}\right) + \cos \left( 2\pi f_\textrm{II} t+\delta _\textrm{II}\right) \sin \left( \beta _\textrm{II}\right) \big ) \bigg ) . \end{aligned} \end{aligned}$$

By comparing the coefficients of Eqs. (16) and (17), we obtain

18 $$\begin{aligned} \begin{aligned} R_\textrm{I} \sin (\beta _\textrm{I})&=\frac{\epsilon }{8}C \sin (\chi ) , \hspace{1cm} R_\textrm{I} \cos (\beta _\textrm{I})=\frac{\epsilon }{8} \left[ 1 + C \cos \left( \chi \right) \right] \\ R_\textrm{II} \sin (\beta _\textrm{II})&=-\frac{\epsilon }{8}C \sin (\chi ) , \hspace{1cm} R_\textrm{II} \cos (\beta _\textrm{II})=\frac{\epsilon }{8} \left[ 1 + C \cos \left( \chi \right) \right] \\ \Rightarrow R^2_\textrm{I}&=R^2_\textrm{II}=R^2(C,\epsilon )=\frac{\epsilon ^2}{8^2}\left[ C^2\sin ^2(\chi )+ \left( 1 + C \cos \left( \chi \right) \right) ^2\right] \\ R_\textrm{I}(C,\epsilon )=R_\textrm{II}(C,\epsilon )=R(C,\epsilon )&=\frac{\epsilon }{8}\sqrt{1+2 C \cos \left( \chi \right) +C^2 }\\ \frac{R_j(C=1,\epsilon =1)}{I_0(C=1)}&=A_{j,\textrm{corr}},\\ \tan \left( \beta _\textrm{I}\right)&=-\tan \left( \beta _\textrm{II}\right) =\frac{ C\sin (\chi )}{1 + C \cos \left( \chi \right) }\\ \beta _j(C,\epsilon )&=\pm \arctan \left( \frac{ C\sin (\chi )}{1 + C \cos \left( \chi \right) }\right) \\ \beta _j(C=1,\epsilon =1)&=\varphi _j, \end{aligned} \end{aligned}$$

by substituting Eq. (5). The data is corrected accordingly by scaling the extracted values as

19 $$\begin{aligned} A_{j,\textrm{corr}}&=\frac{\frac{R_j(C=1,\epsilon =1)}{I_0(C=1)}}{\frac{R_j(C=C_\textrm{exp},\epsilon =\epsilon _\textrm{exp})}{I_0(C=C_\textrm{exp})}} A_{j, \textrm{exp}} \end{aligned}$$

20 $$\begin{aligned} \varphi _{j,\textrm{corr}}&=\frac{\beta _j(C=1,\epsilon =1)}{\beta _j(C=C_\textrm{exp},\epsilon =\epsilon _\textrm{exp})}\varphi _{j, \textrm{exp}} . \end{aligned}$$

## Supplementary Information

Supplementary Information.

## Data Availability

The data that support the findings of this study are available under http://doi.ill.fr/10.5291/ILL-DATA.3-16-14.
