# Supplementary material for: Simultaneous path weak-measurements in neutron interferometry
Source: Sci Rep. 2024 Oct 29;14:25994. doi: 10.1038/s41598-024-76167-6 (PMC11522425; doi:10.1038/s41598-024-76167-6)
Supplement: Supplementary file 1 — Supplementary Information. [file 41598_2024_76167_MOESM1_ESM.pdf]

Supplementary Material: Simultaneous Path Weak-Measurements in Neutron Interferometry

Armin Danner[1], Ismaele V. Masiello[1], Andreas Dvorak[1], Wenzel Kersten[1], Hartmut Lemmel[1,2], Richard Wagner[1], and Yuji Hasegawa[1,3]

[1]*Atominstitut, TU Wien, Stadionallee 2, 1020 Vienna, Austria*

[2]*Institut Laue-Langevin, 71 avenue des Martyrs, 38000 Grenoble, France*

[3]*Department of Applied Physics, Hokkaido University, Kita-ku, Sapporo 060-8628, Japan*

(Dated: October 22, 2024)

Supplementary Note 1: Extended Calculation of the Intensity

$$
\begin{aligned}
&I_{\mathrm{ideal}}(t)\\
&=\left|\langle \mathrm{f}| \left(\hat{U}_{\mathrm{RF}}(t,\alpha,f_{\mathrm{I}},\delta_{\mathrm{I}})\hat{\Pi}_{\mathrm{I}}+\hat{U}_{\mathrm{RF}}(t,\alpha,f_{\mathrm{II}},\delta_{\mathrm{II}})\hat{\Pi}_{\mathrm{II}}\right)|\mathrm{i}\rangle\right|^2\\
&\approx\left|\frac{1}{\sqrt{2}}\left(\langle \mathrm{I}|+\langle \mathrm{II}|\right)\langle\uparrow_x|\left[\begin{pmatrix}1 & \mathrm{i}\frac{\alpha}{2}\mathrm{e}^{+\mathrm{i}(2\pi f_{\mathrm{I}}t+\delta_{\mathrm{I}})}\\ \mathrm{i}\frac{\alpha}{2}\mathrm{e}^{-\mathrm{i}(2\pi f_{\mathrm{I}}t+\delta_{\mathrm{I}})} & 1\end{pmatrix}\hat{\Pi}_{\mathrm{I}}\right.\right.\\
&\quad\left.\left.+\begin{pmatrix}1 & \mathrm{i}\frac{\alpha}{2}\mathrm{e}^{+\mathrm{i}(2\pi f_{\mathrm{II}}t+\delta_{\mathrm{II}})}\\ \mathrm{i}\frac{\alpha}{2}\mathrm{e}^{-\mathrm{i}(2\pi f_{\mathrm{II}}t+\delta_{\mathrm{II}})} & 1\end{pmatrix}\hat{\Pi}_{\mathrm{II}}\right]\frac{1}{\sqrt{2}}\left(|\mathrm{I}\rangle+\mathrm{e}^{-\mathrm{i}\chi}|\mathrm{II}\rangle\right)|\uparrow_z\rangle\right|^2\\
&=\left|\frac{1}{2}\left(\langle \mathrm{I}|+\langle \mathrm{II}|\right)\langle\uparrow_x|\left[|\mathrm{I}\rangle\left(|\uparrow_z\rangle+\mathrm{i}\frac{\alpha}{2}\mathrm{e}^{-\mathrm{i}(2\pi f_{\mathrm{I}}t+\delta_{\mathrm{I}})}|\downarrow_z\rangle\right)+\mathrm{e}^{-\mathrm{i}\chi}|\mathrm{II}\rangle\left(|\uparrow_z\rangle+\mathrm{i}\frac{\alpha}{2}\mathrm{e}^{-\mathrm{i}(2\pi f_{\mathrm{II}}t+\delta_{\mathrm{II}})}|\downarrow_z\rangle\right)\right]\right|^2\\
&=\left|\frac{1}{2}\left(\langle \mathrm{I}|+\langle \mathrm{II}|\right)\frac{1}{\sqrt{2}}\left(\langle\uparrow_z|+\langle\downarrow_z|\right)\left[|\mathrm{I}\rangle\left(|\uparrow_z\rangle+\mathrm{i}\frac{\alpha}{2}\mathrm{e}^{-\mathrm{i}(2\pi f_{\mathrm{I}}t+\delta_{\mathrm{I}})}|\downarrow_z\rangle\right)+\mathrm{e}^{-\mathrm{i}\chi}|\mathrm{II}\rangle\left(|\uparrow_z\rangle+\mathrm{i}\frac{\alpha}{2}\mathrm{e}^{-\mathrm{i}(2\pi f_{\mathrm{II}}t+\delta_{\mathrm{II}})}|\downarrow_z\rangle\right)\right]\right|^2\\
&=\left|\frac{1}{2\sqrt{2}}\left[\left(1+\mathrm{i}\frac{\alpha}{2}\mathrm{e}^{-\mathrm{i}(2\pi f_{\mathrm{I}}t+\delta_{\mathrm{I}})}\right)+\mathrm{e}^{-\mathrm{i}\chi}\left(1+\mathrm{i}\frac{\alpha}{2}\mathrm{e}^{-\mathrm{i}(2\pi f_{\mathrm{II}}t+\delta_{\mathrm{II}})}\right)\right]\right|^2\\
&=\left|\frac{1}{2\sqrt{2}}\left[1+\mathrm{e}^{-\mathrm{i}\chi}+\mathrm{i}\frac{\alpha}{2}\mathrm{e}^{-\mathrm{i}(2\pi f_{\mathrm{I}}t+\delta_{\mathrm{I}})}+\mathrm{i}\frac{\alpha}{2}\mathrm{e}^{-\mathrm{i}(2\pi f_{\mathrm{II}}t+\delta_{\mathrm{II}})}\mathrm{e}^{-\mathrm{i}\chi}\right]\right|^2\\
&\approx\frac{1}{8}\left[1+1+\mathrm{e}^{-\mathrm{i}\chi}+\mathrm{e}^{\mathrm{i}\chi}+\mathrm{i}\frac{\alpha}{2}\mathrm{e}^{-\mathrm{i}(2\pi f_{\mathrm{I}}t+\delta_{\mathrm{I}})}-\mathrm{i}\frac{\alpha}{2}\mathrm{e}^{\mathrm{i}(2\pi f_{\mathrm{I}}t+\delta_{\mathrm{I}})}+\mathrm{i}\frac{\alpha}{2}\mathrm{e}^{-\mathrm{i}(2\pi f_{\mathrm{I}}t+\delta_{\mathrm{I}})}\mathrm{e}^{\mathrm{i}\chi}-\mathrm{i}\frac{\alpha}{2}\mathrm{e}^{\mathrm{i}(2\pi f_{\mathrm{I}}t+\delta_{\mathrm{I}})}\mathrm{e}^{\mathrm{i}\chi}\right.\\
&\quad\left.+\mathrm{i}\frac{\alpha}{2}\mathrm{e}^{-\mathrm{i}(2\pi f_{\mathrm{II}}t+\delta_{\mathrm{II}})}\mathrm{e}^{-\mathrm{i}\chi}-\mathrm{i}\frac{\alpha}{2}\mathrm{e}^{\mathrm{i}(2\pi f_{\mathrm{II}}t+\delta_{\mathrm{II}})}\mathrm{e}^{-\mathrm{i}\chi}+\mathrm{i}\frac{\alpha}{2}\mathrm{e}^{-\mathrm{i}(2\pi f_{\mathrm{II}}t+\delta_{\mathrm{II}})}-\mathrm{i}\frac{\alpha}{2}\mathrm{e}^{\mathrm{i}(2\pi f_{\mathrm{II}}t+\delta_{\mathrm{II}})}\right]\\
&=\frac{1}{8}\left[2\left[1+\cos(\chi)\right]+2\mathrm{Re}\left\{\mathrm{i}\frac{\alpha}{2}\mathrm{e}^{-\mathrm{i}(2\pi f_{\mathrm{I}}t+\delta_{\mathrm{I}})}\right\}+2\mathrm{Re}\left\{\mathrm{i}\frac{\alpha}{2}\mathrm{e}^{-\mathrm{i}(2\pi f_{\mathrm{I}}t+\delta_{\mathrm{I}})}\mathrm{e}^{\mathrm{i}\chi}\right\}+2\mathrm{Re}\left\{\mathrm{i}\frac{\alpha}{2}\mathrm{e}^{-\mathrm{i}(2\pi f_{\mathrm{II}}t+\delta_{\mathrm{II}})}\mathrm{e}^{-\mathrm{i}\chi}\right\}\right.\\
&\quad\left.+2\mathrm{Re}\left\{\mathrm{i}\frac{\alpha}{2}\mathrm{e}^{-\mathrm{i}(2\pi f_{\mathrm{II}}t+\delta_{\mathrm{II}})}\right\}\right]\\
&=\frac{1}{8}\left[2\left[1+\cos(\chi)\right]+2\mathrm{Re}\left\{\mathrm{i}\frac{\alpha}{2}\mathrm{e}^{-\mathrm{i}(2\pi f_{\mathrm{I}}t+\delta_{\mathrm{I}})}+\mathrm{i}\frac{\alpha}{2}\mathrm{e}^{-\mathrm{i}(2\pi f_{\mathrm{I}}t+\delta_{\mathrm{I}})}\mathrm{e}^{\mathrm{i}\chi}+\mathrm{i}\frac{\alpha}{2}\mathrm{e}^{-\mathrm{i}(2\pi f_{\mathrm{II}}t+\delta_{\mathrm{II}})}\mathrm{e}^{-\mathrm{i}\chi}+\mathrm{i}\frac{\alpha}{2}\mathrm{e}^{-\mathrm{i}(2\pi f_{\mathrm{II}}t+\delta_{\mathrm{II}})}\right\}\right]\\
&=\frac{1}{8}\left[2\left[1+\cos(\chi)\right]-2\mathrm{Im}\left\{\frac{\alpha}{2}\mathrm{e}^{-\mathrm{i}(2\pi f_{\mathrm{I}}t+\delta_{\mathrm{I}})}+\frac{\alpha}{2}\mathrm{e}^{-\mathrm{i}(2\pi f_{\mathrm{I}}t+\delta_{\mathrm{I}})}\mathrm{e}^{\mathrm{i}\chi}+\frac{\alpha}{2}\mathrm{e}^{-\mathrm{i}(2\pi f_{\mathrm{II}}t+\delta_{\mathrm{II}})}\mathrm{e}^{-\mathrm{i}\chi}+\frac{\alpha}{2}\mathrm{e}^{-\mathrm{i}(2\pi f_{\mathrm{II}}t+\delta_{\mathrm{II}})}\right\}\right]\\
&=\frac{1}{8}\left[2\left[1+\cos(\chi)\right]-\alpha\mathrm{Im}\left\{\mathrm{e}^{-\mathrm{i}(2\pi f_{\mathrm{I}}t+\delta_{\mathrm{I}})}+\mathrm{e}^{-\mathrm{i}(2\pi f_{\mathrm{I}}t+\delta_{\mathrm{I}})}\mathrm{e}^{\mathrm{i}\chi}+\mathrm{e}^{-\mathrm{i}(2\pi f_{\mathrm{II}}t+\delta_{\mathrm{II}})}\mathrm{e}^{-\mathrm{i}\chi}+\mathrm{e}^{-\mathrm{i}(2\pi f_{\mathrm{II}}t+\delta_{\mathrm{II}})}\right\}\right]\\
&=\frac{1}{8}\left[2\left[1+\cos(\chi)\right]-\alpha\mathrm{Im}\left\{\left(1+\mathrm{e}^{\mathrm{i}\chi}\right)\mathrm{e}^{-\mathrm{i}(2\pi f_{\mathrm{I}}t+\delta_{\mathrm{I}})}+\left(1+\mathrm{e}^{-\mathrm{i}\chi}\right)\mathrm{e}^{-\mathrm{i}(2\pi f_{\mathrm{II}}t+\delta_{\mathrm{II}})}\right\}\right]\\
&=\frac{1+\cos(\chi)}{4}-\frac{\alpha}{8}\mathrm{Im}\left\{\left(1+\mathrm{e}^{\mathrm{i}\chi}\right)\frac{\left(1+\mathrm{e}^{\mathrm{i}\chi}\right)\left(1+\mathrm{e}^{-\mathrm{i}\chi}\right)}{\left(1+\mathrm{e}^{\mathrm{i}\chi}\right)\left(1+\mathrm{e}^{-\mathrm{i}\chi}\right)}\mathrm{e}^{-\mathrm{i}(2\pi f_{\mathrm{I}}t+\delta_{\mathrm{I}})}+\left(1+\mathrm{e}^{-\mathrm{i}\chi}\right)\frac{\left(1+\mathrm{e}^{\mathrm{i}\chi}\right)\left(1+\mathrm{e}^{-\mathrm{i}\chi}\right)}{\left(1+\mathrm{e}^{\mathrm{i}\chi}\right)\left(1+\mathrm{e}^{-\mathrm{i}\chi}\right)}\mathrm{e}^{-\mathrm{i}(2\pi f_{\mathrm{II}}t+\delta_{\mathrm{II}})}\right\}\\
&=\left|\langle \mathrm{f}|\mathrm{i}\rangle\right|^2-\frac{\alpha}{8}\left(1+\mathrm{e}^{\mathrm{i}\chi}\right)\left(1+\mathrm{e}^{-\mathrm{i}\chi}\right)\mathrm{Im}\left\{\mathrm{e}^{-\mathrm{i}(2\pi f_{\mathrm{I}}t+\delta_{\mathrm{I}})}\frac{1}{\left(1+\mathrm{e}^{-\mathrm{i}\chi}\right)}+\mathrm{e}^{-\mathrm{i}(2\pi f_{\mathrm{II}}t+\delta_{\mathrm{II}})}\frac{1}{\left(1+\mathrm{e}^{\mathrm{i}\chi}\right)}\right\}\\
&=\left|\langle \mathrm{f}|\mathrm{i}\rangle\right|^2\left[1-\alpha\mathrm{Im}\left\{\langle\hat{\Pi}_{\mathrm{I}}\rangle_{\mathrm{w}}\mathrm{e}^{-\mathrm{i}(2\pi f_{\mathrm{I}}t+\delta_{\mathrm{I}})}+\langle\hat{\Pi}_{\mathrm{II}}\rangle_{\mathrm{w}}\mathrm{e}^{-\mathrm{i}(2\pi f_{\mathrm{II}}t+\delta_{\mathrm{II}})}\right\}\right]\\
&=\left|\langle \mathrm{f}|\mathrm{i}\rangle\right|^2\left(1-\alpha\left[A_{\mathrm{I}}\sin(\varphi_{\mathrm{I}}-2\pi f_{\mathrm{I}}t-\delta_{\mathrm{I}})+A_{\mathrm{II}}\sin(\varphi_{\mathrm{II}}-2\pi f_{\mathrm{II}}t-\delta_{\mathrm{II}})\right]\right).
\end{aligned}
\tag{S.1}
$$
